# Supplementary material for: A basic macroeconomic agent-based model for analyzing monetary regime shifts
Source: PLoS One. 2022 Dec 22;17(12):e0277615. doi: 10.1371/journal.pone.0277615 (PMC9779001; doi:10.1371/journal.pone.0277615)
Supplement: S1 Table — [18, 28, 29, 30, 42, 43, 45, 47, 50, 51, 54, 56, 63–65, 100]. (PDF) [file pone.0277615.s001.pdf]

Appendix Table 1. Entities and processes shown in Figure 3

Id.	Description	Reference
Basel III	Basel III regulation	BCBS [42]
Bank bailout	Model specification of bailout of insolvent banks	Section 3.5.3
Bankruptcy	Firm bankruptcy model specification	Section 3.3.4
Caiani/ Schasfoort	Credit market components related to Caiani et al. and Schasfoort et al.	Caiani et al. [45] and Schasfoort et al. [28]
Credit demand	Model specification of matching protocol between credit demand and supply	Section 3.5.1
Credit market	Submodel of credit market	Section 3.5
Credit supply	Model specification of credit creation potential	Section 3.5.2
Delli Gatti	Theory based on Delli Gatti and co-authors	Delli Gatti [18]
Dichotomy	Theory of classical dichotomy	
Dosi	Bankruptcy model specification in accordance with Dosi et al.	Dosi et al. [54]
ECB	Monetary Policy Operation Framework according to the European Central Bank	ECB [43,63] and Bindseil [64]
Financial sector	Submodel of credit cycle and fractional reserve system	Section 3.5 and 3.6
Fiscal	Fiscal policy	Section 3.7
GDP EU	Relation of investment and consumption in relation to Gross Domestic Product (GDP)	[51]
Goods demand	Model specification of demand of homogeneous goods	Section 3.3.3
Goods market	Submodel of goods market	3.3
Goods supply	Model specification of supply of homogeneous goods	Section 3.3.1
Gualdi	Goods market components related to Gualdi et al.	Gualdi et al. [47]
Interbank	Model specification of interbank and deposit market	Section 3.6.1
Investment market	Submodel of investment market	Section 3.4
Invest. cycle	Model specification of investment cycle	Section 3.4.1
Invest. demand	Model specification of investment demand	Section 3.4.2
Krug	Interbank components related to Krug	Krug [29]
Labor	Model specification of hire and fire of labor	Section 3.3.2
Mak(h)ro ABM	Final macroeconomic agent-based model. First version: Mak(h)ro_0	
Minsky/ Keynes	Investment theory to Keynes	Minsky [56]
Monetary policy	Model specification of monetary policy	Section 3.6.2
Monetary system	Submodel of fractional reserve system	Section 3.6
Popoyan	Bailout model specification in accordance with Popoyan et al.	Popoyan [30]

Real economy	Submodel of credit cycle and fractional reserve system	Section 3.3 and 3.4
Stock-Flow	Stock Flow Consistency theory	Lavoie and Zezza [100]
Simon	Model specification of price setting in line with bounded rationality	Simon [50]
Taylor	Theory based on Taylor rule	Taylor [65]
