# Supplementary material for: A basic macroeconomic agent-based model for analyzing monetary regime shifts
Source: PLoS One. 2022 Dec 22;17(12):e0277615. doi: 10.1371/journal.pone.0277615 (PMC9779001; doi:10.1371/journal.pone.0277615)
Supplement: S2 Table — (PDF) [file pone.0277615.s002.pdf]

Appendix Table 2. Balance sheet structure of all agent types

The corresponding agent type subscripts are indicated in parentheses in the first column. Balance sheet items have to be interpreted in aggregated form.

**Agent**	**Assets**	**Liabilities**
Households ($_h$)	Bank deposits[a] (Liq_h)	Equity (E_h)
Firms ($_i$)	Bank deposits ($Liq_i + Inv_i$)<br>Inventory (N_i)	Loan liabilities (L_{ib})<br>Interest obligations (I^L_{ib})<br>Equity (E_i)
Banks ($_b$)	Reserves (R_{bc})<br><br>Interest receivables (I^L_{bi})<br>Business loans (L_{bi})<br>Wholesale loans (L^r_{bb})[d]	Deposits<br>(Liq_h; $Liq_i + E_i + Inv_i$)[b]<br>Wholesale liabilities (L^l_{bb})[c]<br>CB liabilities (L_{bc})<br>Equity (E_b)
Central Bank ($_c$)	Loan receivables (L_{cb})	Reserves (R_{cb})<br>Equity (E_{cb})
Government ($_g$)	Liquidity (Liq_g)	Public debt (D_g)<br>Equity (E_g)

[a] Bank deposits of households consists of financial wealth originated from wages or unemployment benefits.
[b] Retail deposits consists of household liquidity; firm liquidity, equity and investment amount.
[c] Interbank loan liabilities (l).
[d] Interbank loan receivables (r).
