# Supplementary figures and images for: A basic macroeconomic agent-based model for analyzing monetary regime shifts
Source: PLoS One. 2022 Dec 22;17(12):e0277615. doi: 10.1371/journal.pone.0277615 (PMC9779001; doi:10.1371/journal.pone.0277615)

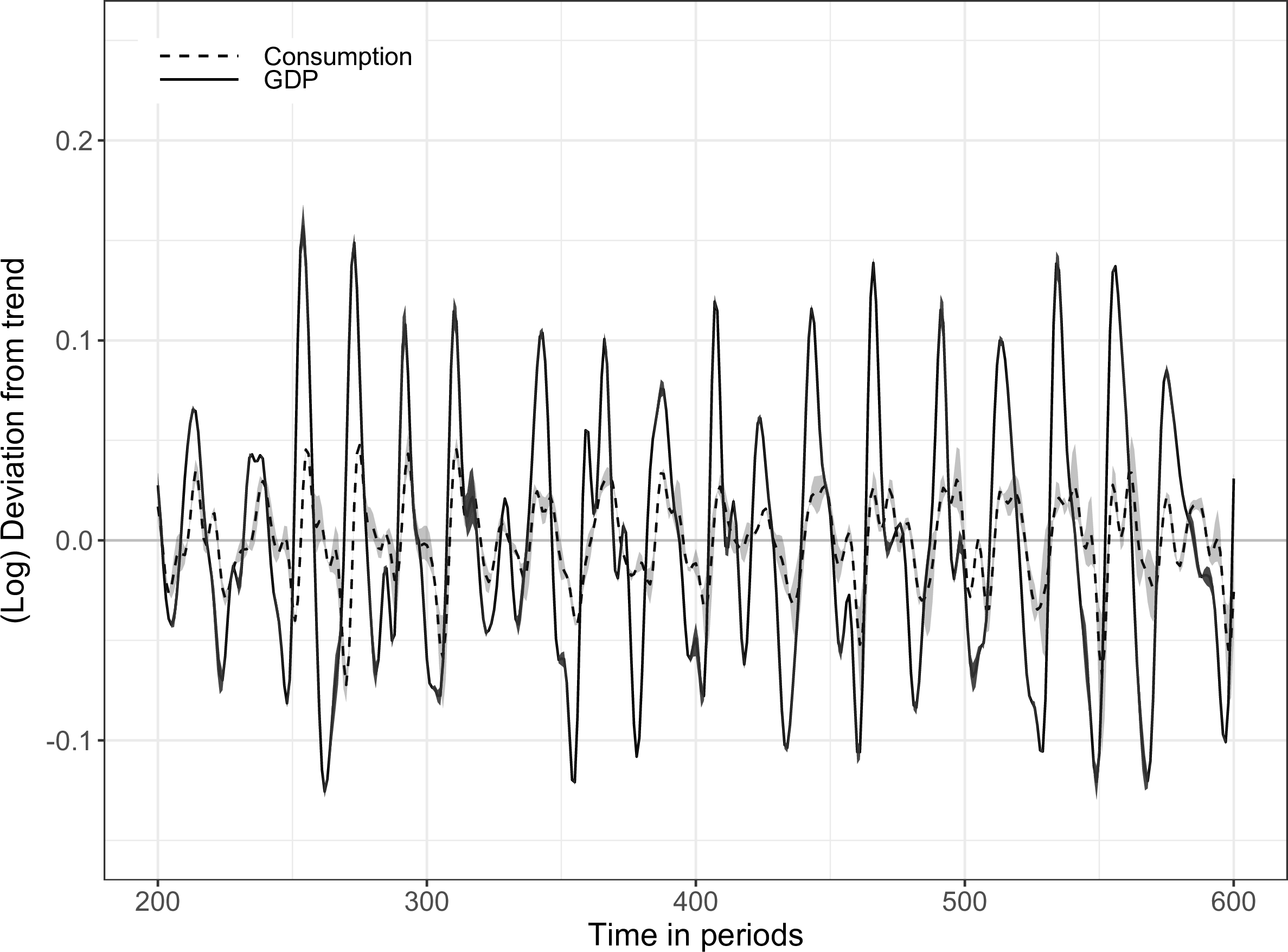

Supplement: S1 Fig — Note: The Figure depicts deviations of average log real GDP (dashed line) and average log real household consumption (solid line) from trend. The simulation output is a representative run and shows an extract to keep the line profile more identifiable without the warm-up phase of 200 simulation periods. The grey shaded area represents the 90% confidence interval. (TIF) [file pone.0277615.s003.tif]
